# Evaluation of Beeswax Influence on Physical Properties of Lipstick Using Instrumental and Sensory Methods

**DOI:** 10.1155/2016/3816460

**Published:** 2016-11-23

**Authors:** Giedre Kasparaviciene, Arunas Savickas, Zenona Kalveniene, Saule Velziene, Loreta Kubiliene, Jurga Bernatoniene

**Affiliations:** Department of Drug Technology and Social Pharmacy, Lithuanian University of Health Sciences, Sukilėlių Pr. 13, LT-50161 Kaunas, Lithuania

## Abstract

The aim of this study was to optimize the lipsticks formulation according to the physical properties and sensory attributes and investigate the relationship between instrumental and sensory analyses and evaluate the influence of the main ingredients, beeswax and oil, with analysis of lipsticks properties. Central composite design was used to optimize the mixture of oils and beeswax and cocoa butter for formulation of lipsticks. Antioxidant activity was evaluated by DPPH free radical scavenging method spectrophotometrically. Physical properties of lipsticks melting point were determined in a glass tube; the hardness was investigated with texture analyzer. Sensory analysis was performed with untrained volunteers. The optimized mixture of sea buckthorn oil and grapeseed oil mixture ratio 13.96 : 6.18 showed the highest antioxidative activity (70 ± 0.84%) and was chosen for lipstick formulation. According to the sensory and instrumental analysis results, optimal ingredients amounts for the lipstick were calculated: 57.67% mixture of oils, 19.58% beeswax, and 22.75% cocoa butter. Experimentally designed and optimized lipstick formulation had good physical properties and high scored sensory evaluation. Correlation analysis showed a significant relationship between sensory and instrumental evaluations.

## 1. Introduction

Safety of various cosmetic products has become a major trend in recent years. The abundant composition of cosmetics is of increasing concern for consumer's health and environmentally friendly products for the earth. Consumers are searching for natural-based cosmetic products to avoid allergic reactions and any side effects and for the safety of their health and the important criteria are natural raw materials [1].

Acceptable lipstick for the consumers should have a suitable texture and spreadability. Descriptive sensory profiling is an essential tool in this process as it allows an experienced panel to assess the qualitative and quantitative characteristics of a product [2]. Hardness and melting point are the main physical properties important for the stability of lipstick in all usage period and transportation. These characteristics can vary according to the composition of ingredients [3]. Therefore, the optimization of mixture composition is important and experimental design is very useful. Statistical mixture design is more satisfactory and effective than other methods such as classical one-at-a-time or mathematical methods because it can study many variables simultaneously with a low number of observations, saving time and costs [4]. In previous works, for the optimization of mixtures, D-optimal cross and mixed designs were used and were quite effective [4]. In this work, response surface central composite design was applied in order to investigate the relationship between composition, physical properties, and sensory analysis of consumers, which can be the most important factor [5].

The common ingredients of lipstick are wax, butter, and oil [6]. Waxes are very useful cosmetic ingredients based on their various advantageous properties. Beeswax is a natural compound secreted by bees and is widely used for dermatological products due to its countless benefits. Beeswax is mainly composed of a mixture of hydrocarbons, free fatty acids, monoesters, diesters, triesters, hydroxy monoesters, hydroxy polyesters, fatty acid polyesters, and some unidentified compounds [7]. This substance contains natural ingredients, which help retain moisture in the skin, especially helpful for dry and chapped lips. Various researches have also discovered that beeswax contains small amounts of natural antibacterial agents and can help prevent a painful inflammation that comes with an infection [8]. Beeswax is vitamin-rich, containing plenty of vitamin A, which helps to improve wound healing, reduces wrinkles, protects the skin against UV radiation, and stimulates skin cells turnover [9]. Natural oils are used in a wide variety of cosmetic products and influence the sensory characteristics of the products [10]. The contents of bioactive lipophilic compounds promote elasticity and healing and moisturize the skin and help it maintain a proper moisture balance and protect the skin from free radical damage. Biologically active compounds of natural oils ensure the beneficial properties of lipsticks: moisturizing and protection from damage of free radicals. There are many scientific lines of evidence on antioxidant, anti-inflammatory, immunomodulatory, regenerative, and other valuable activities of biocompounds of sea buckthorn and grapeseed [11–13]. In this present study, the mixture of buckthorn and grapeseed oils was evaluated for antioxidant activity and used for the lipstick formulation to achieve the highest benefit for the consumers. It was important to evaluate the influence of the main ingredients, beeswax and oil, with analysis of lipsticks properties.

## 2. Materials and Methods

### 2.1. Materials

Yellow beeswax was purchased from Bitute, Lithuania; cocoa butter was purchased from Henry Lamotte GmbH, Germany; jojoba oil, grapeseed (*Vitis vinifera *L.) oil, and sea buckthorn (*Hippophae rhamnoides* L.) oil were from Biokosmetikos Akademija, Lithuania; olive (*Olea europaea *L.) oil was manufactured by Anira, Spain; castor (*Ricinus communis* L.) oil was manufactured by Henry Lamotte Oils, Germany; cedar (*Pinus sibirica*) balm oil was manufactured by Alsu, Russia; essential oil of thyme (*Thymus vulgaris *L.) was manufactured by Aromatika, Ukraine, and was purchased from a local market. Ethanol 96% (Stumbras, Lithuania) and 2,2-diphenyl-1-picrylhydrazyl (DPPH^•^) reagent (Sigma-Aldrich, Germany) were used for the study.

### 2.2. Preparation of Lipsticks

Lipsticks were formulated using a mixture of natural ingredients according to the composition suggested by the central composite response surface design (Table 2). Lipsticks were produced in the laboratory by the moulding method. Beeswax was added to the preheated mixture of oils and heated to 60–67°C until melting. The mixture was homogenized together and cocoa butter was added and melted. The preservative, essential oil of thyme, was added to lipstick base mixture and cooled till 50°C. All of the ingredients were homogenized and poured into clean and lubricated moulds.

### 2.3. Experimental Design

The preliminary formulation of the lipstick was prepared for the suitable lower and upper limits of solid and liquid parts. The results showed that the amount of beeswax 70–30 in percentages and the amount of oil 40–70 in percentages formulate appropriate lipstick. The experimental design is conducted by using Design-Expert® 6 (version 6.0.8, Stat-Ease Inc., Minneapolis, USA). A set of mixtures was formed using response surface design central composite criterion. The experimental mixture design was applied to study the effect of two-component system, amount of oil and amount of beeswax, on the response variables: melting point and the results of sensory analysis. The set of mixtures is shown in Table 2.

### 2.4. Antioxidant Activity

Antioxidant activity was determined by 2,2-diphenyl-1-picrylhydrazyl (DPPH^•^) free radical inactivation method with modifications [14]. For the test, DPPH powders were dissolved in a small volume of ethyl acetate and diluted with ethyl acetate by adjusting the absorbance to 0.700 ± 0.020 at 520 nm. A 20 mg oil sample was weighed in a test tube, and 80 *µ*L ethyl acetate as well as 2.9 mL DPPH^•^ free radical solution was added. The samples were agitated and incubated for 30 min in darkness. Absorbance was measured at 520 nm against ethyl acetate. The percent inhibition of the 2,2-diphenyl-1-picrylhydrazyl (DPPH^•^) free radical was calculated according to the formula

(1) $$\begin{array}{c} {DPPH}^{•}\;\;\left(\;\%\;\;inhibition\;\right)=\frac{\left(A_{blank}-A_{sample}\right)}{A_{blank}}\times 100, \end{array}$$

where *A*_blank_ is the absorbance of the blank solution and *A*_sample_ is the absorbance sample and 2,2-diphenyl-1-picrylhydrazyl (DPPH^•^) free radical solution after 30 min.

### 2.5. Melting Point

Lipstick sample of 2 g was placed into a glass tube. This tube was dipped into a plate full of water, which was heated on the water bath. The temperature at which the material forms a liquid drop was considered its melting point.

### 2.6. Sensory Analysis

Sixty female participants aged 18–25 years who used lip products were selected for sensory evaluation. Filler questions were included so that the applicants could know what the tested lipstick was and their answers would be as honest as possible. Sensory analysis was performed in a room that was well naturally lit with the temperature (25 ± 2°C), humidity (≈55%), and noise controlled. All samples were stored at room temperature (25 ± 2°C) and kept away from direct sunlight. The participants were instructed in the sensory analysis procedure [5]. The bioethics committee of the Lithuanian University of Health Sciences permitted the sensory analysis.

Sensory analysis was performed in three stages before application: initial appearance, during application, and after application (“after texture”). The initial attributes were appearance, colour, and smell. During the application, consistency (hardness), spreadability, and greasiness were evaluated. Ten minutes after the application, the degree of absorption, amount of residue, and moisturizing were evaluated. Thirteen designed lipsticks formulations (Table 2) were evaluated by a panel of female participants. All samples were evaluated in randomized order and lipstick tubes were coded. The panelists filled specific protocols based on 0–10 scale for evaluation of each attribute. Appearance, colour, and smell were scored as follows: 0 means unacceptable and 10 means acceptable. Consistency evaluation scale was as follows: 0 means very soft and 10 means very hard. Spreadability evaluation scale was follows: 0 means very bad and 10 means very good. Greasiness was evaluated as follows: 0 means no feeling and 10 means very greasy. Degree of absorption was scored as follows: 0 means very slow and 10 means very fast. Moisturizing evaluation was as follows: 0 means no feeling and 10 means very good. Amount of residue was scored as follows: 0 means no feeling and 10 means too much. Filled questionnaires were analyzed and profiles of sensory analysis were created for lipsticks comparison.

### 2.7. Texture Profile Analysis

The lipstick hardness is a very important physical characteristic. The texture profile analysis on the formulated products was conducted using a TA.XT.plus (Stable Micro Systems Ltd., Godalming, Surrey, UK) texture analyzer. The penetration depth of a standard 2 mm needle (P/2N) at a constant 5 kg load force was measured to represent the hardness of the lipstick. The sample cuts were placed centrally under the needle probe, which penetrate the sample at 1 mm/s until force of 50 g was achieved. All tests were conducted at room temperature (25 ± 2°C) and repeated three times.

### 2.8. Statistical Analysis

The results are presented as mean ± standard deviation. Statistical analysis was performed by one-way analysis of variance (ANOVA) followed by Tukey's test with the software package Prism v. 5.04 (GraphPad Software Inc., La Jolla, CA, USA). A value of *p* < 0.05 was taken as the level of significance.

## 3. Results

### 3.1. Optimization of Mixture of Natural Oils

**Figure 1:**
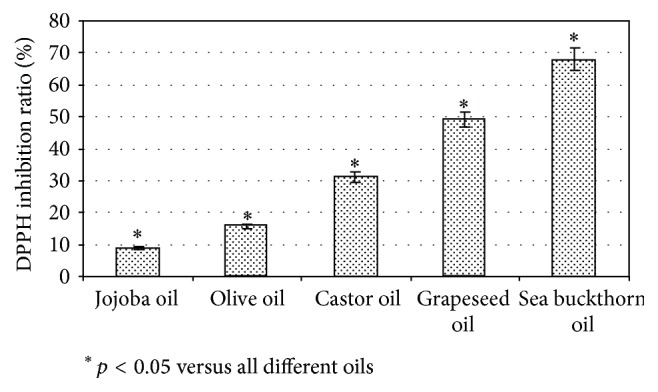
DPPH inhibition percentage of jojoba, olive, castor, grapeseed, and sea buckthorn oils, *n* = 5.

Natural oils of olive, jojoba seed, castor seed, cedar balsam, grapeseed, and sea buckthorn fruits were chosen as potent active constituents for lipstick production. For comparison of their possible antioxidant activities, DPPH free radical scavenging activity was evaluated (Figure 1). The results are expressed as inhibition percentage of free radicals and ranged from 3.92% to 68%. Grapeseed oil and sea buckthorn oil were selected for the lipstick formulation due to their highest inhibition percentage.

Mixture of grapeseed oil and sea buckthorn oil was optimized by central composite design to obtain the highest free radical scavenging effect. The experimental mixture design of the amount of oils and the responses with DPPH inhibition percentages are shown in Table 1.

Response of experimental design with DPPH inhibition percentage ranged from 47.37% to 59.91%. According to the results, it can be concluded that the highest scavenging activity shows mixtures with higher amount of sea buckthorn oil. The target of mixture optimization was the highest DPPH inhibition percentage. To achieve the highest desirability, the amount of oils was changed (from 6 till 14 g). The program designed experimental model and determinations of radical scavenging activity were performed. DPPH scavenging activity ranged from 52.08% to 65.76%. The highest activity was observed in mixture: 14 g of sea buckthorn oil and 6.0 g of grapeseed oil. The lack of fit (*F* value = 0.0352) implies that the model is significant and the optimum mixture was suggested to consist of 13.96 g sea buckthorn oil and 6.18 g grapeseed oil, with the highest desirability of 0.96.

### 3.2. Evaluation of Lipstick Formulations

**Figure 2:**
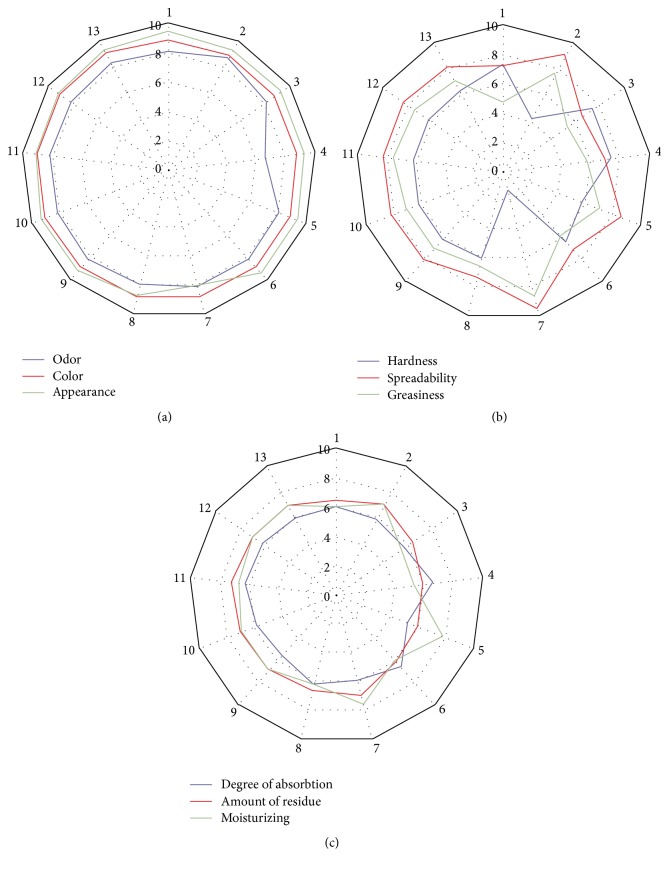
(a) The profile of sensory assessment of the produced lipsticks before application. (b) The profile of sensory assessment of the produced lipsticks during application. (c) The profile of sensory assessment of the produced lipsticks after application.

The experimental design for the lipstick formulations is given in Table 2. Sensorial and physical evaluation was performed as response. Sensory properties of the produced lipsticks were assessed in three stages: before, during, and after application; results are presented in Figures 2(a), 2(b), and 2(c). All tested attributes evaluated of lipsticks are not significantly different: the smell in the acceptability assessment ranged from 6.7 to 8.6 points, while appearance and colour were assessed quite well and ranged from 8.1 to 9.4 points. By analyzing the sensory attributes during application, results showed a more noticeable difference. The consistency varied between 1.3 and 7.6 points, while the desirable parameter was medium at −5 points. The lipstick assessed as the softest consists of 11.89% of beeswax; this was the lowest amount of all formulations. The hardest lipsticks were assessed at −7.4 and 7.4 points and beeswax amount was 30%. All remaining samples were evaluated approximately the same as the average of hardness. It can be concluded that different hardness assessment of lipsticks depends on beeswax amount. Greasiness ranged from 4.7 points to 8.7 points. Medium greasiness was assessed for 5.3 points, which was the desirable assessment, and the amount of oils was medium. The spreadability was assigned to a group of attributes, which should be assessed maximally for 10 points. The highest points were 9.6 points for lipstick consisting of the lowest amount of beeswax and the highest amount of cocoa butter. The lowest assessment was 6.6 points for the lipstick consisting of a medium amount of beeswax, oils, and butter. After application, the amount of residue was the highest for lipstick containing a high amount of butter and a low amount of beeswax. The moisturizing sensory attribute was evaluated very similarly: the highest evaluation for lipsticks containing a high amount of oils and low amounts of beeswax and the lowest evaluation without cocoa butter. The highest degree of absorption was evaluated for lipsticks containing a high amount of oils (70% and 76.21%) and minimum amount of cocoa butter (1.29% and 0%). The highest amount of cocoa butter (43.71%) was associated with the lowest absorption of lipstick. It can be concluded that absorption of lipsticks depends on cocoa butter amount.

Desirable points of sensory evaluation were divided into two groups. Maximum 10 points were for appearance, smell, colour, spreadability, and moistening attributes. Medium 5 points were for hardness, greasiness, amount of residue, and absorption. An average of two groups of assessment points were calculated and used as responses for the mathematical design program.

Another response was the melting points of designed lipsticks (Table 2), which varied from 51.0°C to 69.0°C. The melting point must be high to avoid technical deterioration during preparation and use. Previous studies and researches state the melting point of lipsticks to be in the 60.6–64.0°C acceptable limit by the consumer [4], or else in the 40–56°C range [1]. In this study, the desirable temperature of melting point was set at 60°C.* Design-Expert 6 *program calculated the optimal mixture for the lipstick formulation oil (57.67%), beeswax (19.58%), and cocoa butter (22.75%). The lack of fit (*F* value < 0.0001) implies that the model is significant and the optimum mixture was suggested with the highest desirability of 1.0.

Optimal lipstick formulation of the stated composition was produced and physical parameters were determined: melting point at 59.1°C, when the predicted value was 60°C.

The essential oil of thyme as a natural preservative was added to the final lipstick formulation. It improved smell significantly (*p* < 0.05) while other sensory attributes remained similar (data not shown). Stability studies at one year confirmed that physical parameters remained stable.

**Figure 3:**
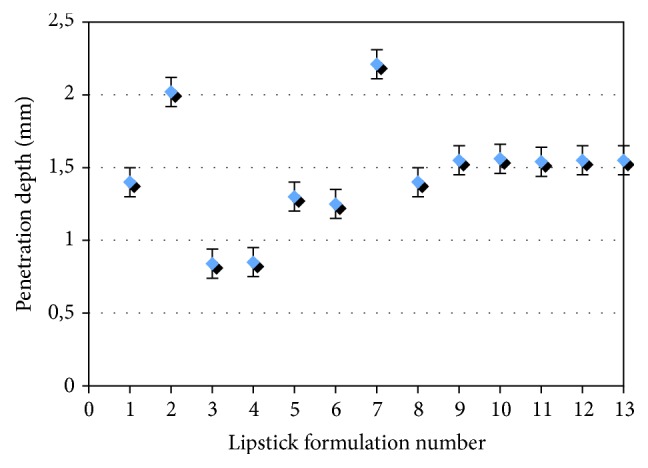
Texture profile penetration depth (mm) for all samples^*∗*^, *n* = 5.

Texture profile analysis was performed to compare sensory evaluation by volunteers and instrumental analysis. Hardness evaluation is an important physical characteristic of lipstick and is a useful tool in objective determination. Assessment points of consistency stated as “hardness” (Figure 2(b)) were compared with penetration depth test. The results of the thirteen designed lipsticks are presented in Figure 3. The penetration depth increases with the increase of oil mixture and decreases with the increase of beeswax. This dependency was observed by other researches also [4, 15].

### 3.3. Correlation Analysis

**Figure 4:**
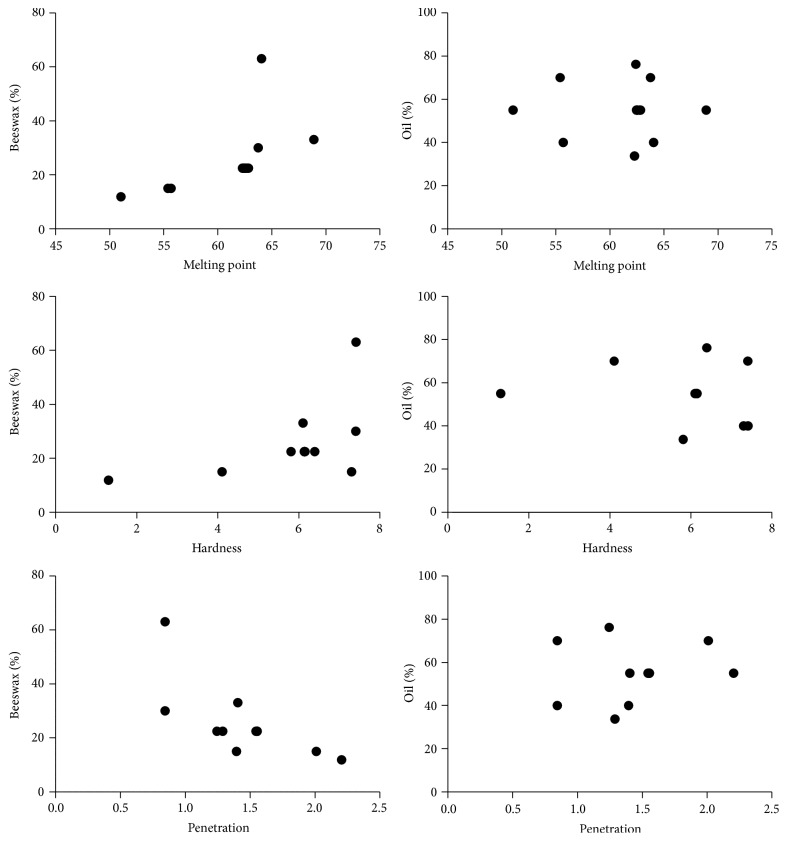
Graphical presentation of correlations of melting point, hardness, and penetration with the amount of beeswax and of oils (mixture of sea buckthorn oil and grapeseed oil in ratio 13.96 : 6.18).

Correlation analysis was performed applying Spearman's rank coefficient. Relationship between hardness, penetration, melting point, and components of lipsticks formulations (beeswax and mixture of oils amounts) was evaluated. Graphical data of correlations is presented in Figure 4. Negative corelation was determined between hardness and penetration depth, *R* = −0.729. High correlation was determined in another study, which showed that the mechanical test (objective method) was in good agreement with the sensory analysis (subjective method) [16]. It can be concluded that sensory analysis and instrumental analysis data are confirmed. Another important characteristic, melting point, correlates with the amount of beeswax (*R* = 0.912), while the amount of oils mixture produces a minimal effect. Amount of beeswax had higher influence on hardness and penetration depth than amount of oils mixture also. Hardness and penetration depth showed medium dependance on the melting point, *R* = 0.484 and *R* = −0.441, respectively. Results of this study correspond to the results of other researches [1].

## 4. Discussion

There are a lot of researches on the health benefits of various vegetable oils depending on their composition and biological activities. In this research, we evaluated the natural oils with the goal of providing the biological activity for the lipstick. Lips are very sensitive to the negative environmental factors and to the damage of free radicals; therefore, it was important to choose natural oil with high antiradical activity. We evaluated six natural oils and the highest results showed grapeseed oil 49,03 ± 0,53% and sea buckthorn oil 68 ± 0,72% of inhibited DPPH radicals. High antioxidant capacity of various oils of grapeseed was determined by other researches and such activity is due to powerful polyphenols constituents like flavan-3-ols and procyanidins [17]. Previous studies revealed that sea buckthorn oil is a potent antioxidant with 73,5 ± 3,39% of DPPH free radicals inactivation and this can be due to the amount of carotenoids and other antioxidants [11, 18].

A good lipstick should have acceptable characteristics for the consumers; therefore, the sensory evaluation was performed. After initial evaluation before use, the mean point was 8.05, which showed that appearance, smell, and colour were well approved. During application of the lipstick, hardness, spreadability, and greasiness were evaluated. Sensory evaluation of consistency varied mostly, from 1.3 to 9.6 points, and the main outcomes are the hardness depending on the amount of beeswax and spreadability depending on the amount of cocoa butter. The feeling after use was divided into three attributes: degree of absorption, moisturizing, and amount of residue. Sensory evaluation results showed that all ingredients were important, but the most influencing ingredient was cocoa butter amount. For comparison with other researches, there are missing published studies, but the main consumers' demand is a natural-based product with good feeling after use.

Instrumental analysis consisted of determination of physical characteristics: melting point and penetration depth. Results of this study show that increasing the amount of beeswax gives a higher value of melting point and smaller value of penetration depth. Previous studies and researches demonstrated that the melting point of lipsticks and the hardness depend on the amount variations of waxes [1, 4, 15]. The influence of beeswax was confirmed as high by correlation analysis.

## 5. Conclusion

Experimental design was used to optimize mixture of grapeseed and buckthorn oils with the highest antioxidative activity and the final lipstick formulation. The optimal ratio of ingredients was assessed to evaluate the sensory and physical properties. Sensory acceptance and instrumental analysis showed that beeswax had higher influence on the lipstick properties, followed by other ingredients. The penetration depth increases with the increase of oil mixture and decreases with the increase of beeswax. Correlation analysis showed a significant relationship between sensory and instrumental evaluations.

## Figures and Tables

**Table 1:** Experimental data of sea buckthorn and grapeseed amounts obtained from mixture design and response, DPPH inhibition percentages of oils mixtures.

Number	Sea buckthorn oil (g)	Grapeseed oil (g)	Response, DPPH inhibition (%)
1	6.00	6.00	53.94 ± 1.10
2	10.00	10.00	59.91 ± 1.15^*∗*^
3	6.00	6.00	54.98 ± 1.10
4	6.00	11.66	57.72 ± 1.12
5	6.00	6.00	52.60 ± 1.10
6	11.66	6.00	59.86 ± 1.15^*∗*^
7	2.00	10.00	47.37 ± 1.06
8	10.00	2.00	54.62 ± 1.11
9	2.00	2.00	51.72 ± 1.10
10	6.00	6.00	56.28 ± 1.12
11	6.00	0.34	52.24 ± 1.10
12	6.00	6.00	55.63 ± 1.10
13	0.34	6.00	48.88 ± 1.10

^*∗*^ *p* > 0.05  *versus* formulation number 6.

**Table 2:** The set of component mixtures for the lipstick formulations and the response, melting point.

Number	Oil mixture, %	Beeswax, %	Cocoa butter^*∗*^, %	Melting point, °C
1	40.00	15.00	45.00	56.0
2	70.00	15.00	15.00	55.0
3	40.00	30.00	30.00	64.0
4	70.00	30.00	0	64.0
5	33.79	22.50	43.71	62.0
6	76.21	22.50	1.29	62.0
7	55.00	11.89	33.11	51.0
8	55.00	33.11	11.89	69.0
9	55.00	22.50	22.50	63.0
10	55.00	22.50	22.50	63.0
11	55.00	22.50	22.50	63.0
12	55.00	22.50	22.50	62.0
13	55.00	22.50	22.50	62.0

^*∗*^Amount of cocoa butter is calculated till 100%.
